# Gut microbiome-derived bacterial extracellular vesicles in patients with solid tumours

**DOI:** 10.1016/j.jare.2024.03.003

**Published:** 2024-03-07

**Authors:** Surbhi Mishra, Mysore Vishakantegowda Tejesvi, Jenni Hekkala, Jenni Turunen, Niyati Kandikanti, Anna Kaisanlahti, Marko Suokas, Sirpa Leppä, Pia Vihinen, Hanne Kuitunen, Kaisa Sunela, Jussi Koivunen, Arja Jukkola, Ilja Kalashnikov, Päivi Auvinen, Okko-Sakari Kääriäinen, T. Peñate Medina, O. Peñate Medina, Juha Saarnio, Sanna Meriläinen, Tero Rautio, Raila Aro, Reetta Häivälä, Juho Suojanen, Mikael Laine, Pande Putu Erawijattari, Leo Lahti, Peeter Karihtala, Terhi S. Ruuska, Justus Reunanen

**Affiliations:** a Research Unit of Translational Medicine, University of Oulu, Oulu, Finland; b Biocenter Oulu, University of Oulu, Oulu, Finland; c Ecology and Genetics, Faculty of Science, University of Oulu, Oulu, Finland; d Research Unit of Clinical Medicine, University of Oulu, Oulu, Finland; e Faculty of Medicine and Health Technology, University of Tampere, Tampere, Finland; f Department of Oncology, Helsinki University Hospital Comprehensive Cancer Center, University of Helsinki, Helsinki, Finland; g FICAN West Cancer Centre and Department of Oncology, Turku University Hospital and University of Turku, 20521 Turku, Finland; h Department of Oncology, Oulu University Hospital, Oulu, Finland; i Finnish Medicines Agency, Tampere, Finland; j Department of Medical Oncology and Radiotherapy and Medical Research Center, Oulu University Hospital and University of Oulu, Oulu, Finland; k Tampere Cancer Center, Faculty of Medicine and Health Technology, Tampere University, Tampere, Finland; l Research Program Unit, Applied Tumor Genomics, Faculty of Medicine, University of Helsinki, Helsinki, Finland; m Cancer Center, Kuopio University Hospital, Northern Savonia Healthcare Municipality, Kuopio, Finland; n Section Biomedical Imaging, Department of Radiology and Neuroradiology and Institute for Experimental Cancer Research, Kiel University, 24105 Kiel, Germany; o Lonza Netherlands B.V., 6167 RB Geleen, the Netherlands; p Translational Medicine Research Unit, Medical Research Center Oulu, Oulu University Hospital, and University of Oulu, Oulu, Finland; q Päijät-Häme Joint Authority for Health and Wellbeing, Department of Oral and Maxillofacial Surgery, Lahti Central Hospital, 15850 Lahti, Finland; r Cleft Palate and Craniofacial Centre, Department of Plastic Surgery, Helsinki University Hospital, 00029 Helsinki, Finland; s Clinicum, Faculty of Medicine, University of Helsinki, 00014 Helsinki, Finland; t Department of Abdominal Surgery, Porvoo Hospital, Hospital District of Helsinki and Uusimaa, Porvoo, Finland; u Department of Computing, University of Turku, Turku, Finland; v Department of Pediatrics and Adolescent Medicine, Oulu University Hospital, Oulu, Finland

**Keywords:** Bacterial extracellular vesicles, Gut microbiota, Cancer, 16S rRNA gene sequencing, Machine learning, Proteome

## Abstract

**Introduction:**

Gut microbiome–derived nanoparticles, known as bacterial extracellular vesicles (bEVs), have garnered interest as promising tools for studying the link between the gut microbiome and human health. The diverse composition of bEVs, including their proteins, mRNAs, metabolites, and lipids, makes them useful for investigating diseases such as cancer. However, conventional approaches for studying gut microbiome composition alone may not be accurate in deciphering host–gut microbiome communication. In clinical microbiome research, there is a gap in the knowledge on the role of bEVs in solid tumor patients.

**Objectives:**

Analyzing the functionality of bEVs using (meta)genomics and proteomics could highlight the unique aspects of host–gut microbiome interactions in solid tumor patients. Therefore, we performed a comparative analysis of the proteome and microbiota composition of gut microbiome-derived bEVs isolated from patients with solid tumors and healthy controls.

**Methods:**

After isolating bEVs from the feces of solid tumor patients and healthy controls, we performed spectrometry analysis of their proteomes and next-generation sequencing (NGS) of the 16S gene. We also investigated the gut microbiomes of feces from patients and controls using 16S sequencing and used machine learning to classify the samples into patients and controls based on their bEVs and fecal microbiomes.

**Results:**

Solid tumor patients showed decreased microbiota richness and diversity in both the bEVs and feces. However, the bEV proteomes were more diverse in patients than in the controls and were enriched with proteins associated with the metabolism of amino acids and carbohydrates, nucleotide binding, and oxidoreductase activity. Metadata classification of samples was more accurate using fecal bEVs (100%) compared with fecal samples (93%).

**Conclusion:**

Our findings suggest that bEVs are unique functional entities. There is a need to explore bEVs together with conventional gut microbiome analysis in functional cancer research to decipher the potential of bEVs as cancer diagnostic or therapeutic biomarkers.

## Highlights

• Gut microbiome-derived bEVs exhibited distinct proteomic changes in solid tumour patients.
• The microbial richness and diversity of both bEVs and total faeces were decreased in patients.
• Bacterial extracellular vesicles appeared as distinct functional entities.
• 16S community structure of faeces-derived bEVs did not resemble the total fecal microbiome.
• The functional potential of gut bEVs in cancer pathogenesis and therapy should be examined.

## Graphical abstract

**Figure:**
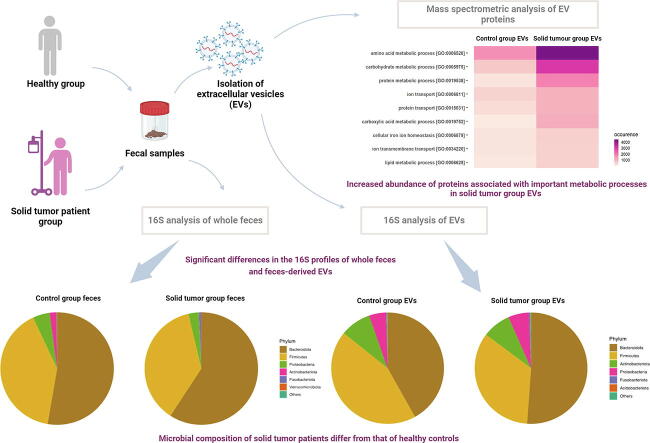

## Introduction

The human gut microbiome is a complex consortium of gut bacteria and their ecological and metabolic collaborations [1]. The number of bacterial cells in the human gut is estimated to be in the same order as the number of human cells [2]. The gut commensal bacteria compete with pathogens; regulate epithelial barrier function, homeostasis, host metabolism; and influence the host’s immune response [3], [4], [5], [6]. Considering that a direct interaction between gut bacteria and the host seems implausible, the extracellular vesicles secreted by these bacteria may play a critical role in achieving this communication. Bacterial extracellular vesicles (bEVs) are membrane-enclosed lipid bilayer nanoparticles containing bacterial molecules, such as lipopolysaccharides, peptidoglycans, nucleic acids, lipids, proteins, and small molecular metabolites, as their cargo [7], [8], [9]. bEVs were first described in Escherichia coli in the 1960s, and research on bEVs has gained substantial momentum in recent decades [10]. Nearly all gut bacteria secrete bEVs for communication between species, survival [11], material exchanges [11], host immune modulation [12], [13], infection, and invasion [14], [15]. bEVs can cross physiological barriers [14], facilitate long‐distance delivery of bacterial active compounds [16] and regulate host cellular responses [17], [18]. Because of these notable properties, bEVs could be utilized as novel tools for the diagnosis and treatment of many human disorders, such as cancer and other diseases [19].

Microbiome-derived extracellular vesicles (EVs) are emerging as a prominent means for studying the connection between gut microbiome communities and human diseases (Supplementary Table 1) [20]. It has been speculated that bEVs derived from the gut microbiome play a plausible role in the tumor development of different types of cancer, including extra-gastric organs [21] and clinical responses to cancer treatment [15], [21], [22], [23], [24]. bEVs derived from *Helicobacter pylori* have been shown to elicit inflammatory mediator production by recipient cells and the development of gastric cancer [25]. *Fusobacterium* nucleatum-derived EVs have been shown to increase the functional activity of human breast cancer cells, such as proliferation, migration, and invasion, *in vitro* [26], providing insight into the role of bacterial EVs in cancer development [27]. Several studies have revealed that EVs secreted by the gram-positive human gut commensal *Bifidobacterium* species are anti-inflammatory, which could benefit tumor development [28], [29]. *Bifidobacterium longum*–derived EVs have been shown to exert anti-inflammatory effects by inducing the secretion of the anti-inflammatory cytokine interleukin-10 (IL-10) from whole splenocytes as well as from cocultures of dendritic cells (DCs) and CD4+ T cells [30].

The metabolomic and genomic profiles of fecal bEVs from colorectal cancer patients have been used to determine the association between colorectal cancer development and the microbiome and metabolic alterations in EV populations. In a clinical study of colorectal cancer patients, Kim et al. reported that gut microbiome–derived EVs encode a dynamic range of metabolic information about the nutritional state of the host, their metabolism, and immune responses in disease conditions [20]. To date, in clinical medicine, gut microbiome- and gut microbiome-derived EVs have been investigated mainly in the context of colorectal cancer [20], [31], [32], [33]. Few studies have investigated gut microbiome–derived bEVs in solid tumor patients as an elemental part of gut microbiome analysis, despite emerging evidence suggesting their involvement in modulating host physiological functions and immune responses in cancer.

Our case-control study is a novel attempt to surpass conventional gut microbiome investigations in clinical research and overcome the technical challenges associated with bEV isolation and characterization. To precisely evaluate the role of bEVs in solid tumor patients, we studied the 16S and proteome profiles of feces-derived bEVs in solid tumor patients and healthy controls, along with their total fecal microbiota profiles. We compared the microbial compositions of solid tumor patients’ feces and bEVs to those of healthy individuals. We also compared the proteomes of gut microbiome–derived bEVs in solid tumor patients and healthy controls. Linking these two omics approaches could enhance the precision of the distinction between solid tumor patients and healthy individuals. Further studies to examine the functional roles of bEVs in the development of solid tumors could help expand their potential as effective diagnostic and/or therapeutic tools.

## Subjects and methods

### Study design

We enrolled 28 solid tumor patients with a mean age of 64.1 years (SD 9.5) and 19 healthy controls with a mean age of 64.8 years (SD 10.5) for this multicenter case-control study conducted in five university hospitals in Finland. We aimed to compare the bEVs in fecal samples drawn from patients with solid cancer tumors and from healthy controls.

### Ethics statement

The present study was conducted according to the ethical policies and procedures approved by the Helsinki University Hospital District Regional Committee on Medical Research Ethics (HUS/1377/2020) and Oulu University Hospital Ethical Committee (EETTMK 12/2020). All study participants gave their written informed consent.

### Clinical characteristics of the patients

The clinical characteristics of the patients are presented in Supplementary Table 2. All cancers were either stage III or stage IV at study entry. All patients were initiating immuno-oncological therapy, and the histological malignancies were malignant melanomas (n = 5), renal cell carcinomas (n = 5), nonsmall cell lung cancers (n = 15), urothelial carcinomas (n = 1), head and neck squamocellular carcinomas (n = 1), and a cancer of unknown origin (n = 1). Patients were recruited at baseline (i.e., before the commencement of antiprogrammed cell death receptor (PD1) or antiprogrammed cell death receptor ligand (PD-L1) therapy), and the samples were collected from June 2020 to February 2021. Seven of the twenty-eight patients reported using antibiotics within three months of the recruitment date. Fecal samples were self-collected by the recruited subjects at home, transported to the laboratory, and stored at −80 °C in sterile cryovials.

### Clinical characteristics of controls

Controls were recruited from patients who had colonoscopies with normal findings. None of the controls reported using antibiotics within three months of the recruitment date. Other exclusion criteria for controls were any significant infection or diarrhea less than six months ago, ulcerative colitis, Crohn’s disease, coeliac disease or another bowel disease, previous cancer, or a change in bowel movement. Fecal samples were self-collected by the recruited subjects at home before bowel preparation for colonoscopy. The collected fecal samples were transported to the laboratory and stored at −80 °C.

### Extraction of extracellular vesicles from fecal samples

Using a method described in a previous study, extracellular vesicles (EVs) were isolated from the fecal samples by employing a combination of filtration, size-exclusion chromatography, and density gradient ultracentrifugation [34]. Fecal samples were thawed on ice, and 1 g of each fecal sample was suspended in 15 ml of sterile 1x PBS and then centrifuged at 14,000 g for 30 min at 4 °C to remove cell debris and undigested impurities. The supernatants were then filtered on ice using a 40 μm nylon filter (Falcon Cell Strainer 352340) and a 0.45 μm polyethersulfone (PES) filter (150 ml vacuum bottle filter 404000, FPE404150) on ice. Purified samples were concentrated in Amicon® Ultra-15 centrifugal filter units (Millipore, #UFC910024) by centrifugation at 3000g for 30 min at 4 °C. The concentrate was collected to isolate EVs using commercial Exo-Spin™ Mini-Columns (Cell Guidance Systems; #EX03). Isolated EVs were purified and enriched for bacterial markers using OptiPrep™ (#115778535; STEMCELL Technologies) density gradient centrifugation. bEVs have been reported to be allocated mostly to gradient fractions 6 and 7, which were used for further downstream analysis [34]. EV preparations were stored at −20 °C until further use. Sterile PBS was used as the starting material for the negative controls for the entire process.

### Transmission electron microscopy analysis

A general assessment of the presence and purity of the purified EVs was conducted using transmission electron microscopy (TEM) [34], [35]. For this purpose, EV samples were negatively stained at the EM Core Facility, Biocenter Oulu. From each sample, 5 μL was deposited into Formvar carbon-coated and glow-discharged copper grids by incubation for 20 min. The sample was then fixed to the grid with 1 % glutaraldehyde, which was followed by negative staining with a 2 % methylcellulose–0.4 % uranyl acetate solution. The grids were observed using a Tecnai G2 Spirit 120-kV transmission electron microscope with Veleta and Quemesa CCD cameras (Tissue Imaging Center, Biocenter Oulu, Oulu, Finland), and images were captured [36].

### Nanoparticle tracking analysis

The concentration and size distribution of the EV samples were determined through nanoparticle tracking analysis (NTA) [34], [35] using Nanosight NM300 with NTA software version 3.4.4. The EV samples were diluted to a factor of 1:100 for optimal measurements. The run script contained four measurement cycles of 60 s each. The final EV concentration was calculated as the mean value of all four runs. Unpaired t-tests were used to compare the mean sizes and concentrations of EVs from healthy controls and solid tumor patients. Statistical analysis was done using GraphPad Prism (version 10.0.2).

### Protein extraction from extracellular vesicles

Protein extraction from EVs was performed using Friedman’s methanol precipitation method [37], with some changes that included the use of SYPRO™ Ruby protein gel stain (Invitrogen™ S12001) and Laemmli loading buffer (4x) as a replacement for CyDyes™. The samples, distilled water, methanol, and chloroform were mixed at a ratio of 1:3:4:1, respectively, and centrifuged at 14,000 g for 1 min to precipitate the protein, with the protein precipitate appearing at the interface. The water–methanol mix at the top of the interface was removed, and the precipitate was washed with four volumes of methanol, pelleted, and dried at room temperature. The dried pellets were resuspended in 1x Laemmli loading buffer and boiled at 95 °C for 5 min. Of each sample, 20 μL was loaded onto 12 % separating gels (Mini-Protean TGX, Bio-Rad), and electrophoresis was run at 110 V for 10–15 min. The gel was then fixed in 50 % ethanol-10 % acetic acid solution for 30 min by shaking at room temperature. The fixed gel was stained with 20 ml of 1x SYPRO™ Ruby protein gel stain in a container covered with aluminum foil at room temperature overnight and then destained with 5 % acetic acid for 5 min in a shaker. After destaining, the gel was incubated in distilled water in a shaker for 15 min. Protein bands were then visualized under UV light, cut, and stored in Eppendorf tubes. The gel pieces were dehydrated using 99 % ethanol and sent for mass spectrometry analysis. PBS controls from EV isolation were used as negative controls for protein isolation and mass spectroscopy analysis [36].

### Mass spectrometry (MS) and proteomic analysis of fecal extracellular vesicles

The MS analysis of the EV proteins was performed by the Turku University proteomics facility in Turku, Finland. For this purpose, samples were subjected to liquid chromatography-electrospray ionization-tandem mass spectrometry (LC-ESI-MS/MS) analysis on a nanoflow high performance liquid chromatography (HPLC) system (Easy-nLC1200, Thermo Fisher Scientific, Bremen, Germany) attached to a Q Exactive HF mass spectrometer (Thermo Fisher Scientific) with a nanoelectrospray ionization source. Peptides were loaded on a trapping column and then separated on a 15 cm C18 column (75 μm × 15 cm, ReproSil-Pur 3 μm 120 Å C18-AQ; Dr. Maisch HPLC GmbH, Ammerbuch-Entringen, Germany). The mobile phase consisted of solvent A (water with 0.1 % formic acid) and solvent B (acetonitrile/water (80:20 v/v) with 0.1 % formic acid). A linear 20 min gradient from 8 % to 43 % of solvent B was used to elute the peptides. Thermo Xcalibur 4.1 software (Thermo Fisher Scientific) was used to collect the MS data. The information-based acquisition method entailed an Orbitrap MS survey scan with a mass range of 300–2,000 *m*/*z*, followed by high-energy collision dissociation fragmentation for the 10 peptide ions with the highest intensity [36].

Proteomics on MS data was performed using Peaks Studio software (version 10.6) [38]. Proteins were identified by searches in the UniProt Swissprot and UniProt trEMBL (UniProt release 2022_05) databases. Parent mass error tolerance was fixed at 10.0 ppm, and fragment mass error tolerance was set at 0.02 Da. A false discovery rate (FDR) of 1.0 % was fixed for both peptide and protein identification [38]. Proteins represented by at least one unique peptide and the total protein coverage from supporting peptides greater than or equal to 1 % were identified. Only the top proteins with the most assigned hits from each protein group were selected and reported. Negative controls were also analyzed, and protein identifications from the negative controls were excluded from the sample data. Taxonomic names were reported as they appeared in UniProt release 2023_03. Figures were drawn using RStudio (2023.03.1 + 446 for Windows) and R 4.3.0 using the ggplot2 package (version 3.4.2) [36].

### Total RNA extraction from extracellular vesicles

RNA isolation from EVs for conversion to cDNA and use in 16S ribosomal ribonucleic acid (rRNA) gene sequencing was carried out using an exoRNAeasy Serum Plasma Midi Kit (Qiagen) according to the manufacturer’s instructions. A phenol/guanidine-based combined lysis and elution step was implemented, followed by silica membrane–based purification to recover total RNA. High-quality RNA was eluted in a small volume of RNase-free water [39].

### Conversion of RNA to cDNA

RNA isolated from the EV samples was converted to cDNA using the iScript cDNA synthesis kit (Bio-Rad) according to the manufacturer’s instructions. Thereafter, 2 µL (approximately 20 ng) of RNA template, 2 µL (final concentration 0.5 µM) of 16S rRNA specific primer 515F (5′-GTGCCAGCMGCCGCGGTAA-3′), and 1x iScript reaction buffer were used in the reaction mixture [39].

### DNA extraction from fecal samples

A QIAamp Fast DNA Stool Mini Kit (Qiagen) was used to isolate high-quality purified DNA from fecal samples for polymerase chain reaction (PCR) and 16S rRNA gene sequencing, with 180–220 mg of fecal sample used as the starting material. Lysis and separation of impurities from fecal samples were performed using InhibitEX Buffer, and the DNA was purified on QIAamp Mini spin columns [40]. The extracted DNA integrity and fragment size were determined using 1 % agarose gel electrophoresis. The concentrations of the extracted DNA were measured using NanoDrop 2000.

### Polymerase chain reaction (PCR) amplification and 16S rRNA gene sequencing

The V4–V5 hypervariable region of the 16S gene was sequenced using the S-*-Univ-0519-a-S-18 (5́CAGCMGCCCGCGGTAATWC-3′) and S-D-Bact-0907-a-A-20 (5′-CCGTCAATTCCTTTRAGTTT-3′) primers. The forward primer contained an individual 9 bp barcode to enable multiplexing of samples in the sequencing run. Amplification was done in duplicate 15-μL PCR reactions according to the manufacturer’s protocol using Phusion Flash High Fidelity PCR master mix (Thermo Scientific), 10 ng of fecal DNA, and 0.5 μM of forward and reverse primers. The sequencing run also included two negative controls, along with two mock community controls of HM-782D and Microbial Mock Community B. Amplification was performed with a Biosystems™ Veriti 96-Well Thermal Cycler (Thermo Scientific) with an initialization phase of 3 min at 98 °C, 22 cycles of amplification with an annealing temperature of 64 °C, and a final elongation phase of 5 min at 72 °C [39].

After amplification, duplicate reactions were combined, and the presence of PCR products was confirmed using agarose gel electrophoresis. For sequencing, the PCR products were purified using AMPure XP (Beckman Coulter, CA, USA) reagent; the concentration was measured with Bioanalyser DNA-1000 chips, and equivalent amounts of products for each sample were pooled together. Multiplexed samples were further purified using AMPure XP reagent and analyzed with Bioanalyser DNA-1000 chips; the final DNA concentrations were measured with a Quant-iT PicoGreen dsDNA Assay Kit (Thermo Fisher Scientific). Sequencing was performed with an Ion Torrent PGM sequencer using an Ion PGM Hi-Q View template kit (400 bp templating program), an Ion PGM Hi-Q View sequencing kit (850 cycles), and a 316 v2 chip [36], [39].

### 16S rRNA sequence analysis

The 16S rRNA sequence analysis used Quantitative Insights into Microbial Ecology 2 (QIIME2; version 2022.2) [41]. Reads shorter than 200 bp were removed. Selected reads were demultiplexed and denoised using QIIME2-implemented divisive amplicon denoising algorithm 2 (DADA2) [42], and chimeric reads were removed. The subsequent step was trimming reads at base 30 and truncation at base 330, depending on the quality of the plots produced by QIIME2 in the demultiplexing step. Bacterial taxa were assigned to representative sequences using the SILVA database version 138.1 [43]. Taxa characterized as mitochondria, eukaryota, cyanobacteria, and archaea were excluded from further analysis. Negative controls and the R *decontam* package (version 1.8.0) were used to filter out the contaminant reads using the prevalence-based method with a threshold of 0.5 [44]. The reads in the processed data had a mean frequency of 12,385 and 12,874 and a median frequency of 11,526 and 10,906 in the EVs and fecal samples, respectively. Reads were rarefied with a sampling depth of 4,092 for EVs and 4,316 for fecal samples.

Chao1, Shannon index, Simpson index, and observed richness were used to estimate within-sample diversity, which is known as the alpha diversity. The statistical significance of alpha diversity differences was calculated using the Kruskal–Wallis H test, with a *p*-value of 0.05 or less as the significance threshold. Between-sample diversity—known as beta diversity—was evaluated by performing principal coordinate analysis (PCoA) using the Bray–Curtis dissimilarity index. Rarefaction was performed for data normalization, here based on the total read counts, with the lowest read count serving as the threshold for the rarefaction process. The statistical significance of the beta diversity results was tested using PERMANOVA [45]. Analysis of the composition of the microbiomes (ANCOM) was performed using QIIME2 to identify differentially abundant taxa between the cases and controls. Differential abundance was represented by the W value [46]. Visualization of alpha and beta diversity was performed in RStudio (version 2023.03.0 + 386) using the microeco package (version 0.15.0).

### Metadata classification

To validate the taxonomic differences between the healthy control and solid tumor groups, machine learning analysis was performed to determine the possibility of clarifying whether the sample was taken from a healthy control or solid tumor patient. We used the taxonomy data produced during the 16S analysis for this purpose. Analysis was performed separately for EV samples derived from feces and whole fecal samples. The random forest nested cross-validation (NCV) algorithm was applied using QIIME2, and the number of estimators was set at 150 [41.].

## Results

### Transmission electron microscopy

TEM was performed to confirm the presence of EVs and visualize their morphology. Distinctive amounts of vesicles were observed in both the healthy control and solid tumor patient samples. The morphology of the vesicles resembled the nanosized bilayer membrane structures that are typical of bacterial membrane vesicles (Supplementary Fig. 1). The bEV-enriched fractions (6 and 7) were found to be pure (i.e., free from impurities such as flagella, debris, or fibers) and deemed fit for further downstream processing. EV preparations of sterile PBS were used as negative controls for imaging.

### Nanoparticle tracking analysis

We performed NTA to determine the concentrations and size distributions of the isolated EVs. The mean (SD) size of EVs from the solid tumor cohort was 202 (26.8) nm, whereas the mean size of EVs from healthy controls was 168.9 (5.8) nm (mean difference 95 % CI [-61.12, −5.381], *p* = 0.021). The mean (SD) concentration of EVs isolated from solid tumor patients was 1.08 x10^8^ particles/mL (SD 0.77 × 10^8^) and that of EVS isolated from healthy controls 2.52 x10^8^ particles/mL (SD 2.15 × 10^8^) (mean difference 95 %, CI [0.3456, 2.526], *p* = 0.011) (Supplementary Fig. 2).

### Proteomic analysis of fecal extracellular vesicles

The UniProtKB trEMBL and UniProtKB Swissprot databases were used to identify proteins in feces-derived EVs. We identified 23,758 peptides from UniProtKB trEMBL and 7,935 peptides from UniProtKB Swissprot. Altogether, 7,184 bacterial and 1,945 human proteins were identified from healthy control EVs. EVs from solid tumor patients represented a total of 9,115 bacterial proteins and 1,936 human proteins.

**Fig. 1:**
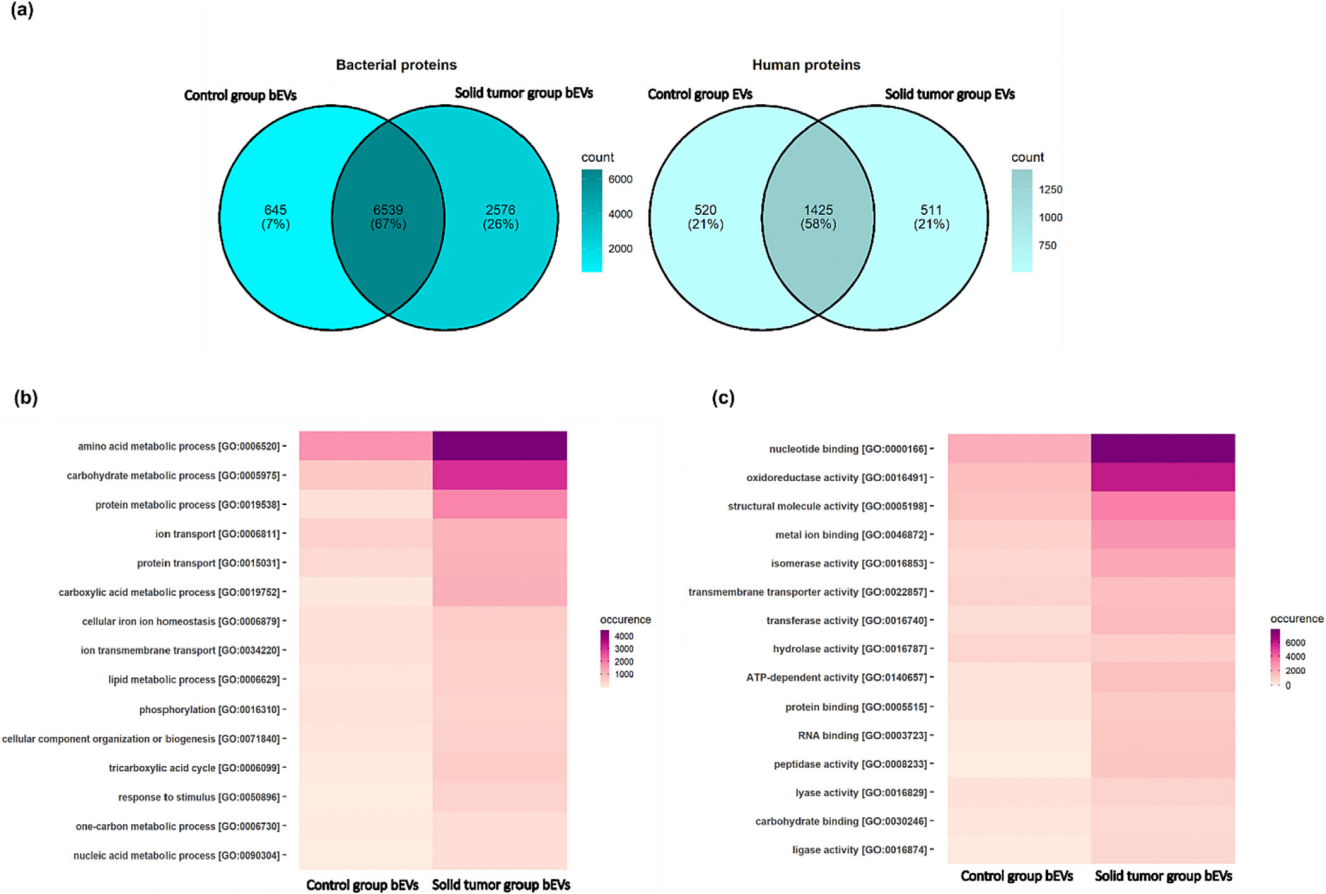
Classification of proteins identified in the gut-derived extracellular vesicles (EVs) from solid tumor patients and healthy controls using UniProtKB trEMBL and UniProtKB Swissprot databases. (a) Venn diagram representing the distribution of bacterial and human proteins in gut-derived EVs in healthy controls and solid tumor patients. Color scale represents the protein counts in the EVs from respective study groups (b) Top 15 GO biological process classes of bacterial proteins identified in gut-derived bacterial extracellular vesicles (bEVs) in healthy controls and solid tumor patients (c) Top 15 GO molecular function classes of bacterial proteins identified in gut-derived bacterial extracellular vesicles (bEVs) in healthy controls and solid tumor patients. Color scale represents the occurrence of a GO class in protein identification.

We then compared the protein counts in EVs derived from solid tumor patients to those from healthy controls. EVs from solid tumor patients had 2,576 unique bacterial proteins, whereas only 645 bacterial proteins were unique to EVs from healthy controls. A total of 6,539 bacterial proteins were shared between the EVs of the preceding two cohorts. EVs from both study groups had 1,425 human proteins in common. The number of human proteins unique to solid tumor patient EVs was 511, whereas healthy control EVs had 520 human proteins (Fig. 1a).

Bacteroidetes, Firmicutes, Proteobacteria, and Actinobacteria were the bacterial phyla with the most frequently assigned protein hits in the gut microbiome–derived bEVs of the healthy controls and solid tumor patients. Proteins belonging to the phyla Bacteroidetes, Firmicutes, and Proteobacteria were more abundant in the bEVs of the solid tumor patients compared with the controls (Supplementary Fig. 3).

Gene ontology (GO) annotations were used to classify the bacterial proteins in the bEVs of solid tumor patients and healthy controls based on their biological processes and molecular functions. GO annotations for biological processes were available for 22.5 % of the proteins from the bEVs of the solid tumor group and for 11.3 % of proteins from the bEVs of the healthy control group. For molecular function, GO annotations were available for 28.4 % of the proteins in the bEVs of solid tumor patients and for 18.3 % of the proteins in the bEVs of the healthy controls. There was an increase in the bacterial protein classes for biological processes, such as the amino acid metabolic process [GO:0006520], carbohydrate metabolic process [GO:0005975], protein metabolic process [GO:0019538], carboxylic acid metabolic process [GO:0019752], and protein transport process [GO:0015031], in the bEVs of solid tumor patients compared with those of healthy controls (Fig. 1b). Nucleotide binding [GO:0000166], oxidoreductase activity [GO:0016491], structural molecule activity [GO:0005198], metal ion binding [GO:0046872], and transmembrane transporter activity [GO:0022857] were the chief bacterial protein classes for molecular function enriched in the bEVs of solid tumor patients compared with the bEVs of healthy controls (Fig. 1c). The GO enrichments based on manual annotation for biological processes and molecular functions are reported in Supplementary Table 3.

### Microbiota composition of gut microbiome-derived bEVs

**Fig. 2:**
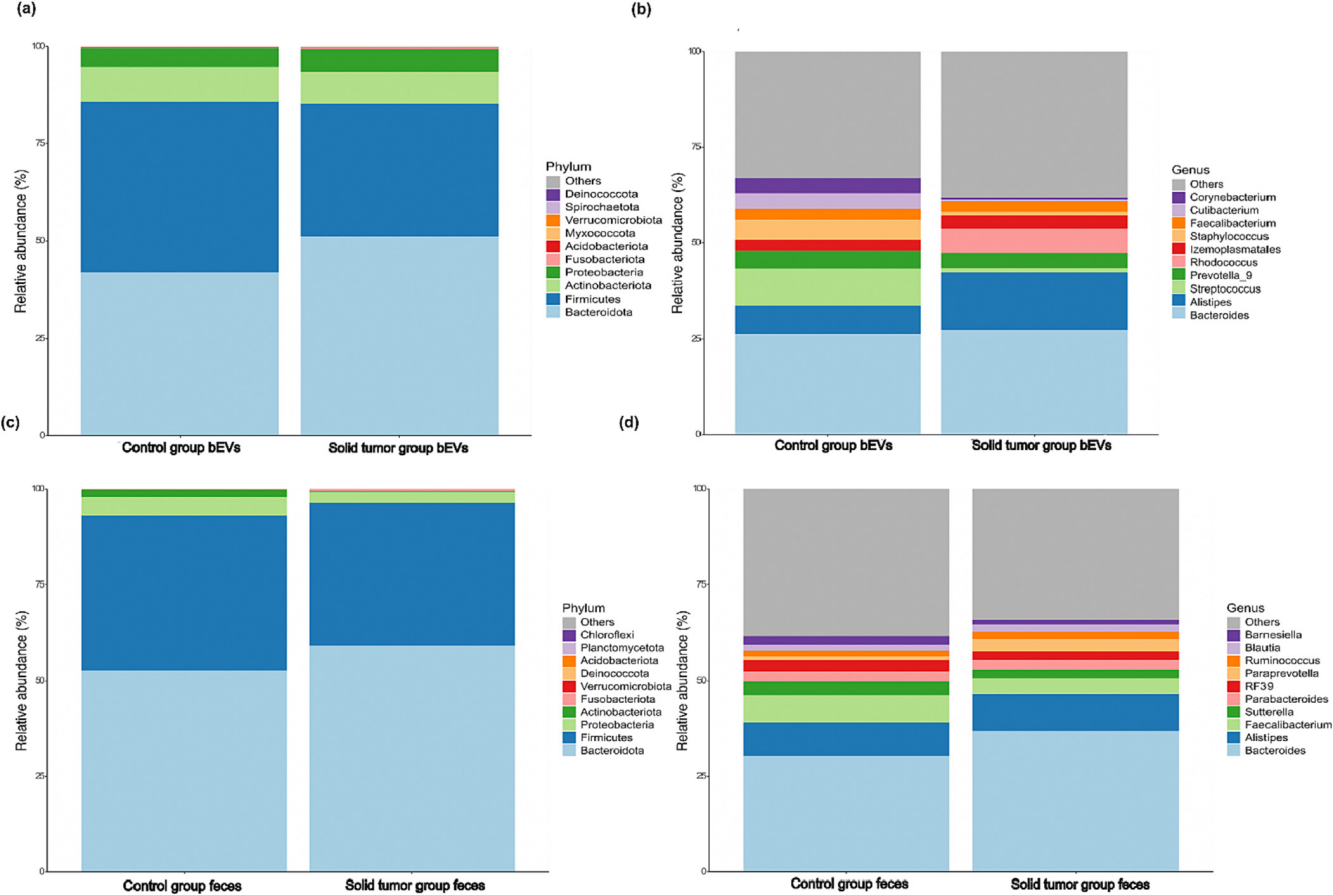
Bar graphs representing relative abundance of bacterial taxa at phylum and genus levels, obtained from 16S rRNA sequence analysis of fecal bEVs and whole feces using QIIME2. The relative abundance of bacterial taxa differs noticeably between healthy controls and patients. (a) Most abundant bacterial phyla in gut microbiome-derived bacterial extracellular vesicles (bEVs) of healthy controls and solid tumor patients (b) Most abundant bacterial genera in gut microbiome-derived bacterial extracellular vesicles (bEVs) of healthy controls and solid tumor patients (c) Most abundant bacterial phyla in the whole feces microbiota of healthy control and solid tumor patients (d) Most abundant bacterial genera in the whole feces microbiota of healthy control and solid tumor patients.

From the bEVs of solid tumor patients and healthy controls, 16S rRNA gene sequences were obtained, and the composition of their gut microbiota was compared at the phylum and genus levels. Solid tumor patients exhibited an increased relative abundance of phyla Bacteroidota (51.1 %), Proteobacteria (5.9 %), and Fusobacteriota (3.5 %). Firmicutes (43.9 %) and Actinobacteria (8.9 %) were comparatively more abundant in healthy controls (Fig. 2a). At the genus level, *Bacteroides* (27.4 %), *Alistipes* (14.9 %)*, Izemoplasmatales* (3.4 %)*,* and *Rhodococcus* (6.4 %) were enriched in the bEVs of solid tumor patients, whereas *Streptococcus* (9.5 %)*, Prevotella* (4.6 %)*,* and *Staphylococcus* (5.3 %) were the major genera found in the bEVs of healthy controls (Fig. 2b). The 10 most abundant taxa in the bEVs of solid tumor patients and healthy controls are reported in Supplementary Table 4a.

The ANCOM of the bEVs of healthy controls and solid tumor patients revealed distinctively abundant bacterial taxa, such as *Rhodococcus*, *Lactobacillus*, *Anaerococcus*, *Streptococcus*, and *Cutibacterium* (Table 1a). The W values provided a measure of the statistical significance of these differences, with higher values suggesting greater significance.

**Table 1:** ANCOM representing differential abundance of bacterial taxa, analysed using QIIME2 (a) Differentially abundant bacterial taxa in the gut microbiota-derived bacterial extracellular vesicles (bEVs) of healthy controls and solid tumor patients (b) Differentially abundant bacterial taxa in the total fecal microbiota of healthy controls and solid tumor patients. The larger the value of W, the more likely the taxon is differentially abundant between controls and patients.

(a) Differentially abundant bacterial taxa bEVs in healthy controls and solid tumor patients						
Taxa						**W value**
Domain	**Phylum**	**Class**	**Order**	**Family**	**Genus**	
Bacteria	Actinobacteriota	Actinobacteria	Corynebacteriales	Nocardiaceace	*Rhodococcus*	441
Bacteria	Firmicutes	Bacilli	Lactobacillales	Lactobacillaceae	*Lactobacillus*	422
Bacteria	Firmicutes	Clostridia	Peptostreptococcales-Tissierellales	Family XI	*Anaerococcus*	418
Bacteria	Firmicutes	Bacilli	Lactobacillales	Streptococcaceae	*Streptococcus*	417
Bacteria	Firmicutes	Actinobacteria	Propionibacteriales	Propionibacteriaceae	*Cutibacterium*	410

(b)Differentially abundant bacterial taxa in the feces of healthy controls and solid tumor patients						
Taxa						**W value**
Domain	**Phylum**	**Class**	**Order**	**Family**	**Genus**	

Bacteria	Bacteroidota	Bacteroidia	Bacteroidales	Prevotellaceace	*Prevotella_7*	276
Bacteria	Bacteroidota	Bacteroidia	Bacteroidales	Prevotellaceace	*Paraprevotella*	253

**Fig. 3:**
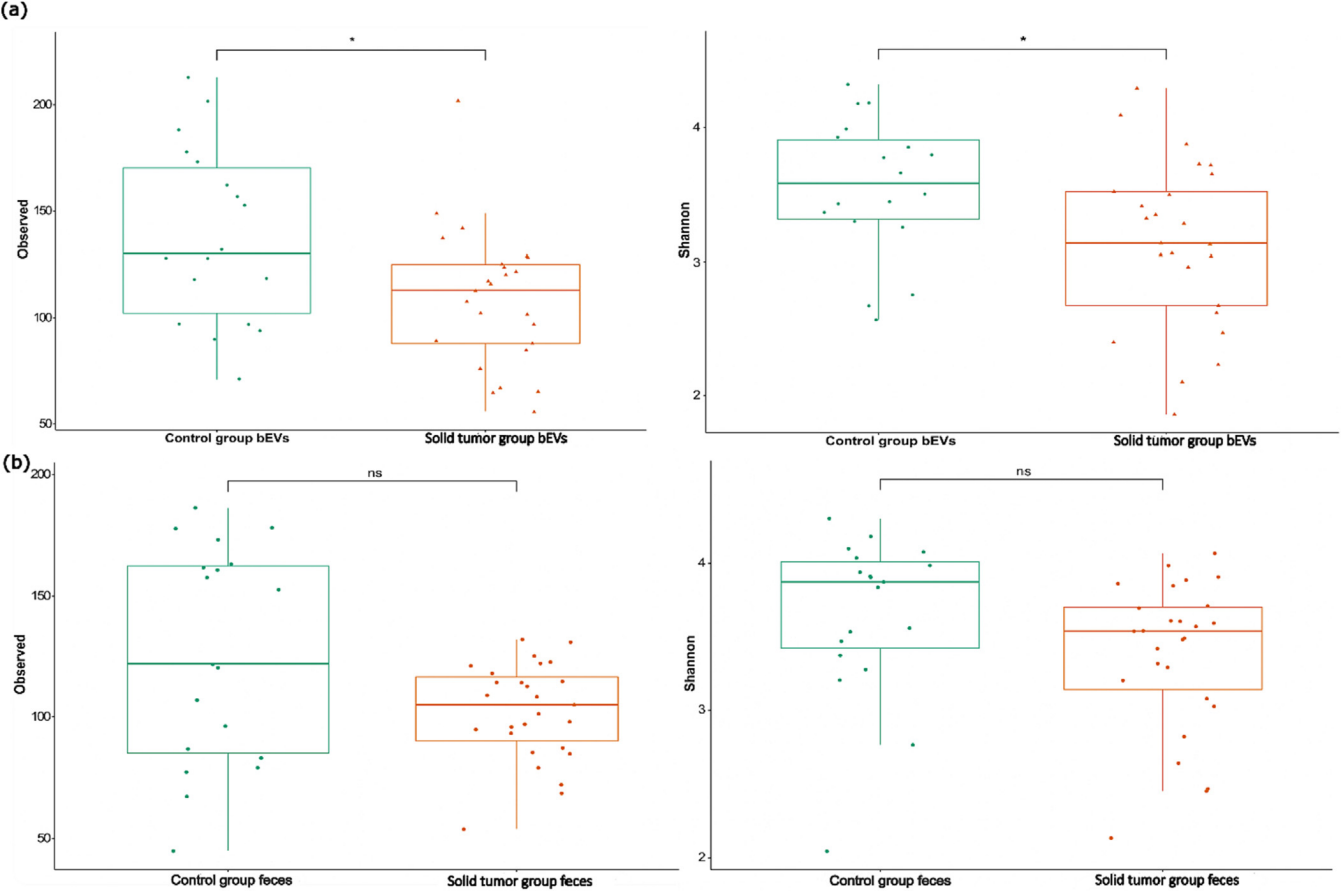
Box plots depicting alpha diversity of gut microbiome-derived bacterial extracellular vesicles (bEVs) and whole feces, analyzed by Observed richness and Shannon index. Solid tumor patients show a decrease in bacterial richness and diversity, as observed in both bEVs and feces. (a) Alpha diversity and richness of gut microbiome-derived bacterial extracellular vesicles (bEVs) of healthy controls vs solid tumor patients (b) Alpha diversity and richness of total fecal microbiota of healthy controls vs solid tumor patients (ns: *p* > 0.05; *: *p* < 0.05).

**Fig. 4:**
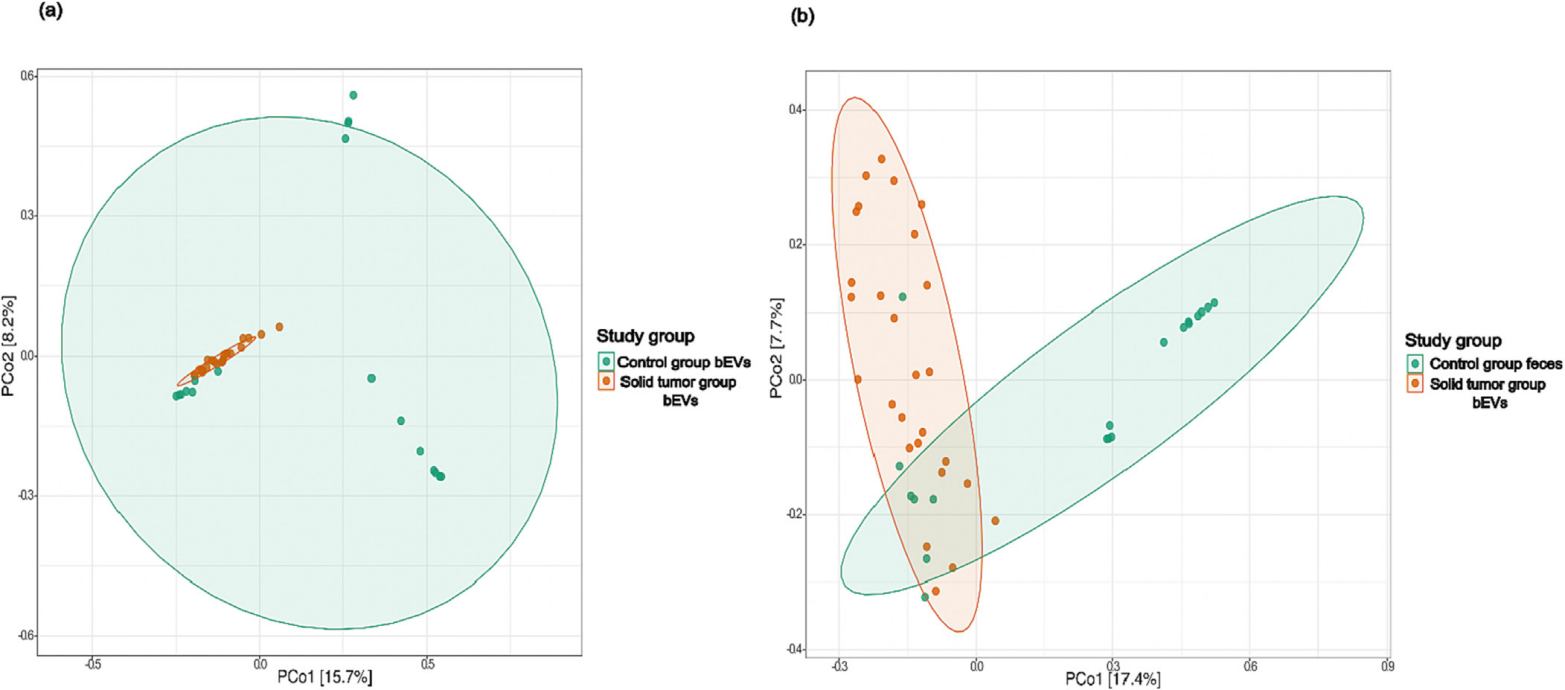
Principal coordinate analysis (PCoA) plots of beta diversity showing clustering of gut microbiome-derived bacterial extracellular vesicles (bEVs) and total fecal microbiota in healthy controls and solid tumor patients. (a) Gut microbiome-derived bacterial extracellular vesicles (bEVs) of healthy controls vs solid tumor patients (b) Total fecal microbiota of healthy controls vs solid tumor patients. Between-sample dissimilarities were measured by Bray Curtis. Permutational multivariate analysis of variance (PERMANOVA) was performed to analyze the statistical significance (*p* = 0.001).

The gut microbiota community structure of bEVs demonstrated substantial differences in richness and diversity between solid tumor patients and control subjects. The within-sample diversity and observed richness were significantly higher in the bEV samples from healthy controls than in those from solid tumor patients (Fig. 3a). Beta diversity analysis showed significant differences between the two sample groups. PCoA with the Bray–Curtis dissimilarity index showed distinct clustering of the solid tumor and healthy control groups. The differences in community composition between the study groups were statistically significant (*p* = 0.001; PERMANOVA). The bEVs of solid tumor patients were closely assembled, implying similarity in microbiota composition, but the bEVs of the healthy controls exhibited more heterogeneous profiles, indicating microbiota variability among the samples (Fig. 4a).

### Total fecal microbiota composition in solid tumor patients and healthy controls

We analyzed fecal samples from solid tumor and healthy control groups to compare their gut microbiota composition at the phylum and genus levels. In solid tumor patients, there was a higher relative abundance of the phyla Bacteroidota (59.2 %), and Fusobacteriota (0.6 %), while Firmicutes (40.3 %), Proteobacteria (4.9 %), and Actinobacteria (1.9 %) were more abundant in the healthy controls (Fig. 2c). The genera *Bacteriodes* (36.9 %), *Alistipes* (9.6 %), and *Paraprevotella* (3.2 %) were more abundant in solid tumor patients, whereas healthy controls had a higher relative abundance of *Fecalibacterium* (7.3 %) and *Sutterela* (3.6 %) (Fig. 2d). The 10 most abundant taxa in the feces from solid tumor patients and healthy controls are reported in Supplementary Table 4b. The ANCOM of total fecal microbiome in healthy controls and solid tumor patients revealed distinctively abundant bacterial taxa, pointing to significant variations in the prevalence of *Prevotella* and *Paraprevotella* between the healthy control and solid tumor patient groups (Table 1b).

The richness and diversity of the gut microbiota community was lower in solid tumor patients than in healthy controls. However, the differences in alpha diversity between these two groups were not statistically significant (Fig. 3b) (Supplementary Fig. 4b). PCoA was performed with the Bray–Curtis dissimilarity index to determine the dissimilarity in gut microbiota composition between solid tumor patients and healthy controls. Fecal samples from solid tumor patients and healthy control groups formed two largely distinct but partially overlapping clusters. Thus, solid tumor patients and healthy controls appeared to be two discrete populations with respect to their microbiota composition. The statistical significance of these differences was confirmed by PERMANOVA (*p* = 0.001) (Fig. 4b).

### Metadata classification

**Fig. 5:**
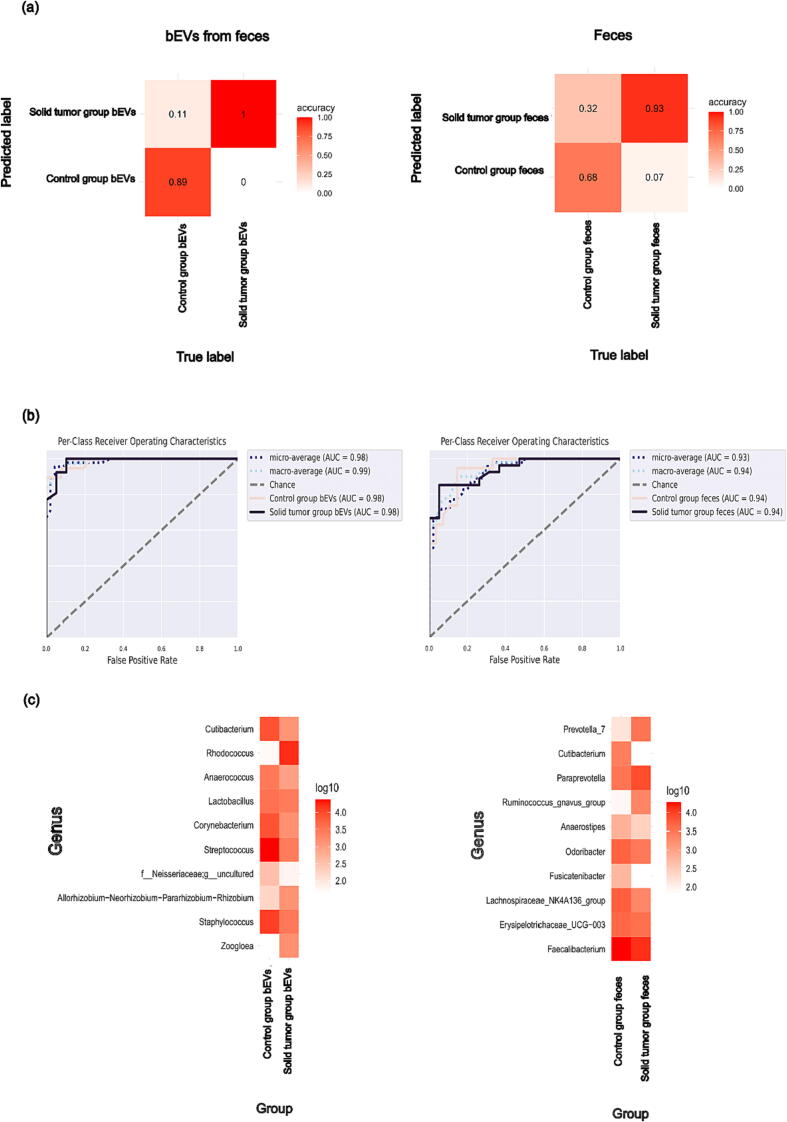
Metadata classification: Random Forest NCV classifying bEV samples from feces (left) and fecal samples (right) into control samples and solid tumor samples. (a) The overall accuracy of the classifier (b) ROC curves of the healthy controls and solid tumor samples (c)The 10 most important bacterial genera used for predicting the sample group with absolute abundances per group as log10 transformations in the order of importance from top to bottom.

Machine learning analysis using random forest NCV was accurate at classifying samples, with 100 % accuracy for fecal bEV sample cases and 93 % for fecal sample cases (Fig. 5a). The algorithm was more prone to false-positive results because both bEVs and fecal control samples were more often classified as solid tumor samples (0.11 and 0.32, respectively) than the other way around (0 and 0.07, respectively). In receiver operating characteristic (ROC) curves, the area under the curve (AUC) was high, in bEVs 0.98 for both controls and cases and in feces 0.94 for both controls and cases (Fig. 5b). Fig. 5c depicts the 10 most important genera for classifying the sample origin. *Cutibacterium* and *Rhodococcus* were the prominent bacterial genera classifying bEVs, whereas *Prevotella* and *Cutibacterium* were the main bacterial classifiers for feces.

## Discussion

In the present study, patients with solid tumors showed decreased gut microbiota richness and diversity in both gut microbiomes and in nanoparticles secreted by gut microbiomes. The proteomes of bacterial EVs, however, were more diverse in patients than in controls, with enrichment of protein classes involved in nucleotide binding, oxidoreductase activity, and metabolism of amino acids and carbohydrates. Thus, even though the community diversity of bacterial EVs was reduced in patients with solid tumors, an increase in the functional (i.e., proteome) level was observed. Given the previously demonstrated abilities of bEVs to facilitate inter/intrakingdom communication, material exchanges, and host immune modulation [47], our results suggest that bEVs may be an underutilized resource in gut microbiome research on cancer patients and could be explored further to address a variety of unmet needs in clinical medicine.

MS analysis of the bEV proteomes of solid tumor patients showed an increased abundance of the proteins involved in amino acid metabolism, carbohydrate metabolism, and protein metabolism, along with the enrichment of proteins associated with molecular functions, such as nucleotide binding, oxidoreductase activity, and structural molecule activity. Metabolism is altered in cancer [48], [49], as exemplified by increased glucose uptake to support cell proliferation and cancer progression, an enhanced rate of glutaminolysis, additional demand for amino acids, increased fatty acid synthesis, and other fluctuations in normal metabolic processes [50], [51]. Our findings suggest that gut microbiome-derived bEVs may encode valuable information regarding metabolic changes and immune responses in solid tumor patients. Further functional studies are required to ascertain the contribution of bEVs to these metabolic changes.

Through 16S rRNA sequencing of gut microbiome-derived bEVs and total feces, we found that the richness and diversity of gut microbiota were relatively lower in solid tumor patients than in healthy controls. In addition, the microbial composition of gut microbiome-derived bEVs was distinct from the total fecal microbiota. bEVs from solid tumor patients were majorly represented by the phyla Bacteroidota, Proteobacteria, and Fusobacteriota, whereas their total fecal microbiome showed a higher relative abundance of Bacteriodota and Fusobacteriota compared with the healthy controls. Firmicutes and Bacteroidota are the primary inhabitants of a healthy gut microbial community, while Proteobacteria, Actinobacteria, and Verrucomicrobia are generally less abundant [20]. Members of Proteobacteria are commensal bacteria with pathogenic potential and inflammatory properties [52]. The increased abundance of Proteobacteria in solid tumor patients in our study may indicate inflammation of the gastrointestinal tract.

At the genus level, bEVs from solid tumor patients were represented by higher relative abundances of *Bacteroides, Alistipes*, *Izemoplasmatales*, and *Rhodococcus,* while the feces of solid tumor patients showed higher relative abundances of the bacterial genera *Bacteroides*, *Alistipes* and *Paraprevotella*. The gut bacterium *Alistipes* is linked to inflammation and colorectal cancer and has previously been shown to trigger tumorigenesis in mice [53], [54]. Some species of *Bacteroides* have been associated with the pathogenesis of colorectal cancers [55]. *Rhodococcus* is an opportunistic bacterium affecting immunocompromised cancer patients [56] and has emerged as an effective diagnostic biomarker in early cancer screening [55]. *Paraprevotella spp*. have been found to preserve intestinal homeostasis by increasing the autolysis of intestinal protease trypsin [57]. Increased levels of trypsin in the intestine have been linked to intestinal pathological conditions such as colitis, Crohn’s disease, and inflammatory bowel disease (IBD) [58], [59], [60]. The order *Izemoplasmatales* has not been much investigated. Decreased richness of ileal mucosal bacteria *norank_f__norank_o__Izemoplasmatales* may have a potential role in the development of hypercholesterolemia in humans [61]. Metadata classification, which is based on bEV and fecal microbiome, was successful in classifying the samples into cases and controls. bEVs were classified more accurately than feces. Some case control studies analyzing the metagenomic profiles of fecal microbiome and bEVs have also implied that the analysis of bEVs has an edge over stool microbiome when it comes to comprehending differences between patients and healthy controls [20], [62].

Our study is a novel effort toward integrating bEVs into conventional gut microbiome research and combining omics methods for studying human health and disease. Our study has several strengths. We used an effective method that was optimized by our research group for the isolation of bEVs from fecal samples. We concurrently used a combination of robust methods, such as TEM, NTA, MS, 16S rRNA sequencing, and machine learning, for the characterization of gut microbiome-derived bEVs, which has not been done in previous studies. In addition, this was a controlled cohort study, which reduced the likelihood of bias in the outcomes. Moreover, the differences between the bEVs of solid tumor patients and healthy controls were statistically significant, despite the limited number of participants. Given the limitations, it is important to emphasize that this was the first step toward investigating bEVs in patients with solid tumors. To better understand the role of gut-microbiome-produced bEVs in the context of treatment efficacy and patient survival, the sample size needs to be increased, and further patient follow-up is required.

## Conclusion

The bEVs of gut commensal bacteria constitute active biological cargo and are essential in assisting in the interactions between host and gut bacteria, highlighting their significant impact on host health [63]. The results of our study indicate that gut microbiota–derived bEVs are distinct taxonomic entities from the parent gut microbiota. This underlines the importance of expanding studies on gut microbiome–derived bEVs in clinical cancer research, along with traditional gut microbiome analysis. Advanced comprehensive functional studies in this direction could help decipher the role of gut microbiome–derived bEVs in cancer pathophysiology and establish a cause-and-effect relationship.

## Funding

This study was funded by Academy of Finland grants 328768 and 299749, Biocenter Oulu, European Regional Development Fund A76179 and Oulu University Hospital.

## Data sharing statement

The mass spectrometry proteomics data have been deposited to the ProteomeXchange Consortium via the PRIDE [1] partner repository with the dataset identifier PXD047510. The raw 16S-rRNA sequences were submitted to GenBank under the accession number PRJNA1020741 for gut microbiota and PRJNA1020742 for extracellular vesicles.

## CRediT authorship contribution statement

**Surbhi Mishra:** Investigation, Formal analysis, Visualization, Writing - Original Draft; **Mysore Vishakantegowda Tejesvi:** Formal analysis, Visualization, Writing - Review & Editing; **Jenni Hekkala:** Formal analysis, Visualization, Writing - Review & Editing; **Jenni Turunen:** Formal analysis, Visualization, Writing - Review & Editing; **Niyati Kandikanti:** Investigation; **Anna Kaisanlahti:** Formal analysis, Visualization, Writing - Review & Editing; **Marko Suokas:** Investigation; **Sirpa Leppä:** Resources; **Pia Vihinen:** Resources, Writing - Review & Editing; **Hanne Kuitunen:** Resources; **Kaisa Sunela:** Resources, Writing - Review & Editing; **Jussi Koivunen:** Resources; **Arja Jukkola:** Resources; **Ilja Kalashnikov:** Conceptualization; **Päivi Auvinen:** Resources; **Okko-Sakari Kääriäinen:** Resources; **T. Peñate Medina:** Writing - Review & Editing; **O. Peñate Medina:** Writing - Review & Editing; **Juha Saarnio:** Resources; Sanna Meriläinen: Resources; **Tero Rautio:** Resources; **Raila Aro:** Resources; **Reetta Häivälä:** Resources; **Juho Suojanen:** Writing - Review & Editing; **Mikael Laine:** Writing - Review & Editing; **Pande Putu Erawijattari:** Writing - Review & Editing; **Leo Lahti:** Writing - Review & Editing; **Peeter Karihtala:** Conceptualization, Resources, Writing - Review & Editing; **Terhi S. Ruuska:** Conceptualization, Supervision, Funding acquisition, Writing - Review & Editing; **Justus Reunanen:** Conceptualization, Supervision, Funding acquisition, Writing - Review & Editing.

## Declaration of competing interest

The authors declare that they have no known competing financial interests or personal relationships that could have appeared to influence the work reported in this paper.
