# Supplementary material for: Gut microbiome-derived bacterial extracellular vesicles in patients with solid tumours
Source: J Adv Res. 2024 Mar 7;68:375–86. doi: 10.1016/j.jare.2024.03.003 (PMC11785572; doi:10.1016/j.jare.2024.03.003)
Supplement: Supplementary data 1 [file mmc1.pdf]

SUPPLEMENTARY INFORMATION

Table of contents:

1. Supplementary Figure 1
2. Supplementary Figure 2
3. Supplementary Figure 3
4. Supplementary Figure 4
5. Supplementary Table 1
6. Supplementary Table 2
7. Supplementary Table 3 (a, b, c, and d)
8. Supplementary Table 4

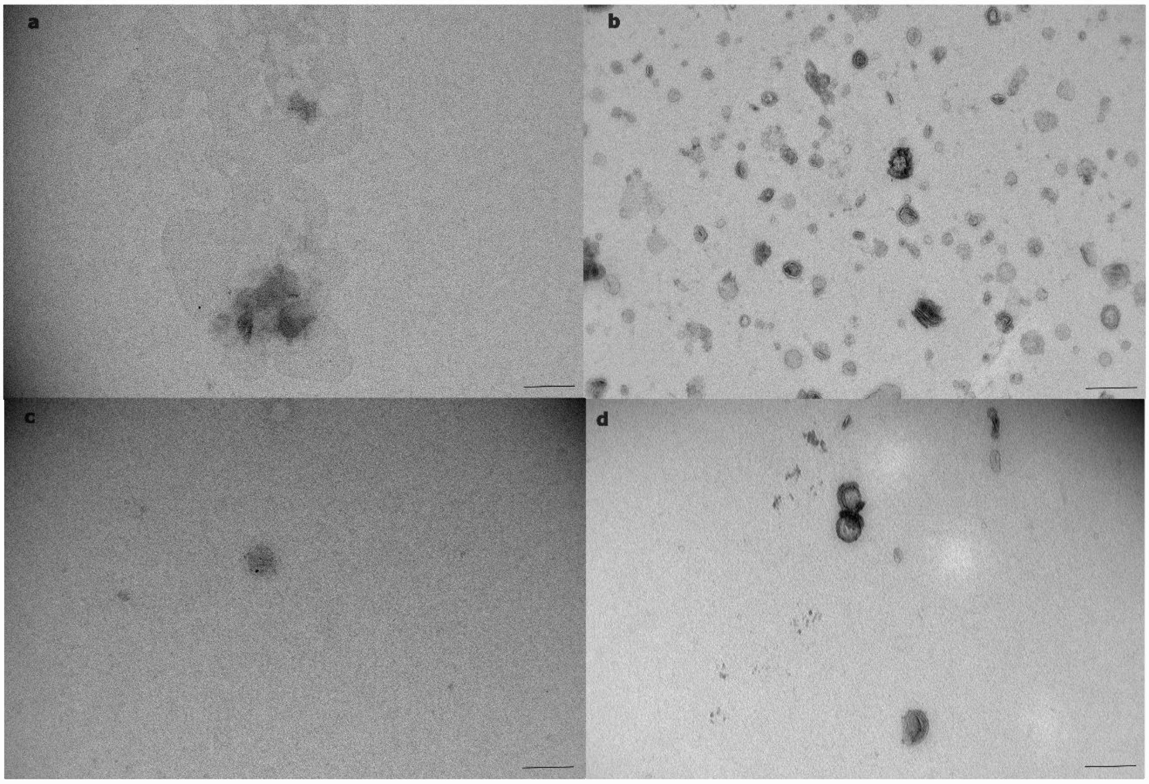

Supplementary Figure 1: Representative photomicrographs of negative stain transmission electron microscopy images (TEM; 23000 x magnification) of fecal EV preparations. (a) & (c) Negative controls, EV isolation from PBS; (b) EV isolation from the feces of solid tumor patients (d) EV isolation from the feces of healthy controls. The size of the scale bar is 200 nm.

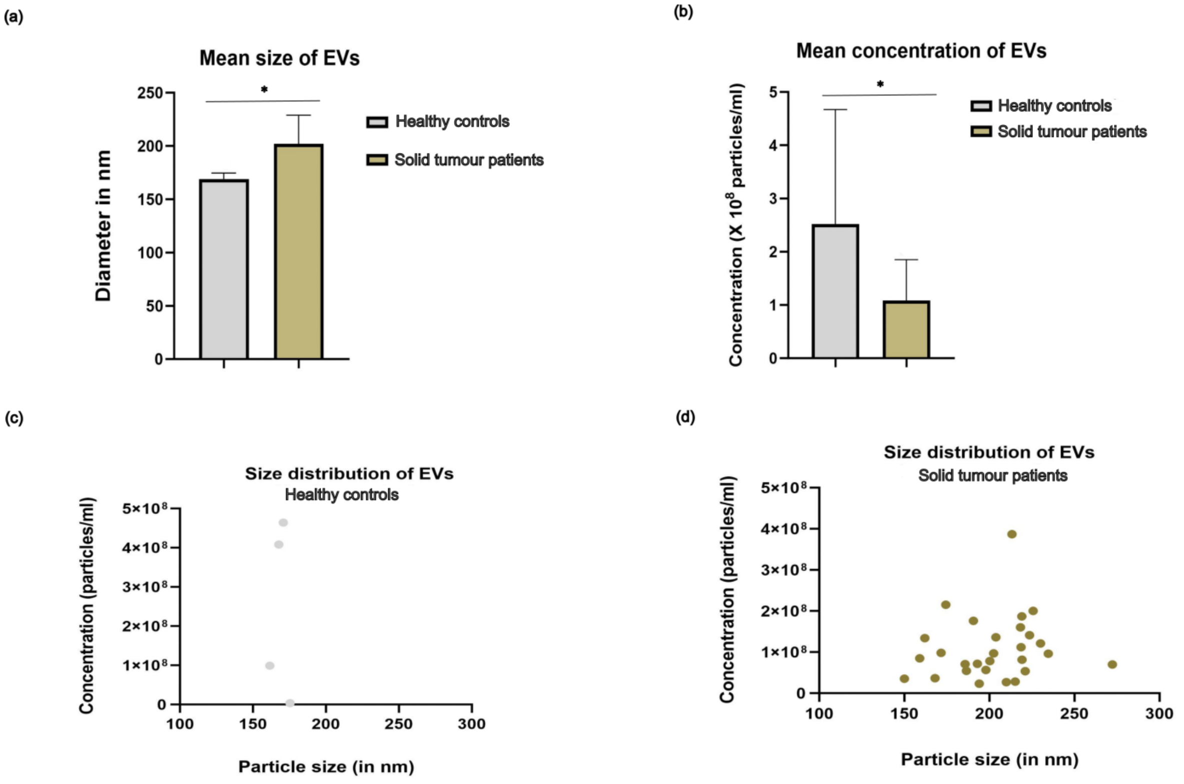

Supplementary Figure 2: Results from Nanoparticle tracking analysis (NTA) of extracellular vesicles (EVs) derived from healthy controls and solid tumour patients. (a) Unpaired t-test comparing mean sizes of the EVs from two study groups (b) Unpaired t-test comparing mean concentrations of the EVs from the two study groups. Error bars represent mean (SD) values in each study group. (c) Size distribution of bEVs from healthy controls (d) Size distribution of bEVs from solid tumour patients. * $p < 0.05$

(a)

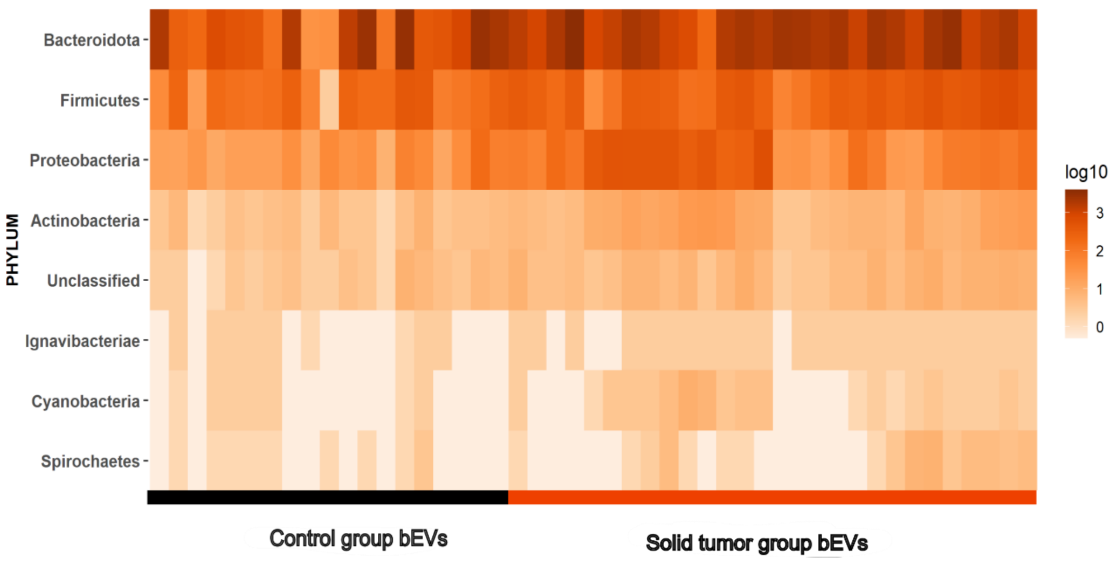

(b)

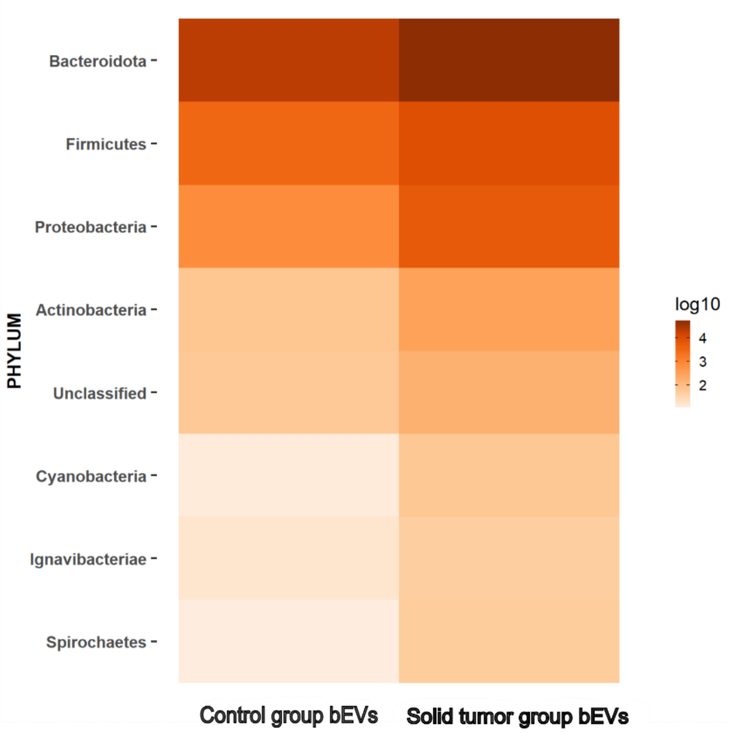

Supplementary Figure 3: Heatmaps representing major protein-producing bacterial phyla, identified from the gut microbiome-derived bacterial extracellular vesicles (bEVs) of healthy controls and solid tumor patients using UniProtKB trEMBL and UniProtKB Swissprot databases. (a) Most abundant protein-producing phyla per control and patient sample (b) Most abundant protein-producing phyla for the entire healthy control and solid tumor patient cohorts. The number of protein hits assigned to each phylum in each sample are presented as log10 score (n = 28 for solid tumour patients; n = 19 for healthy controls).

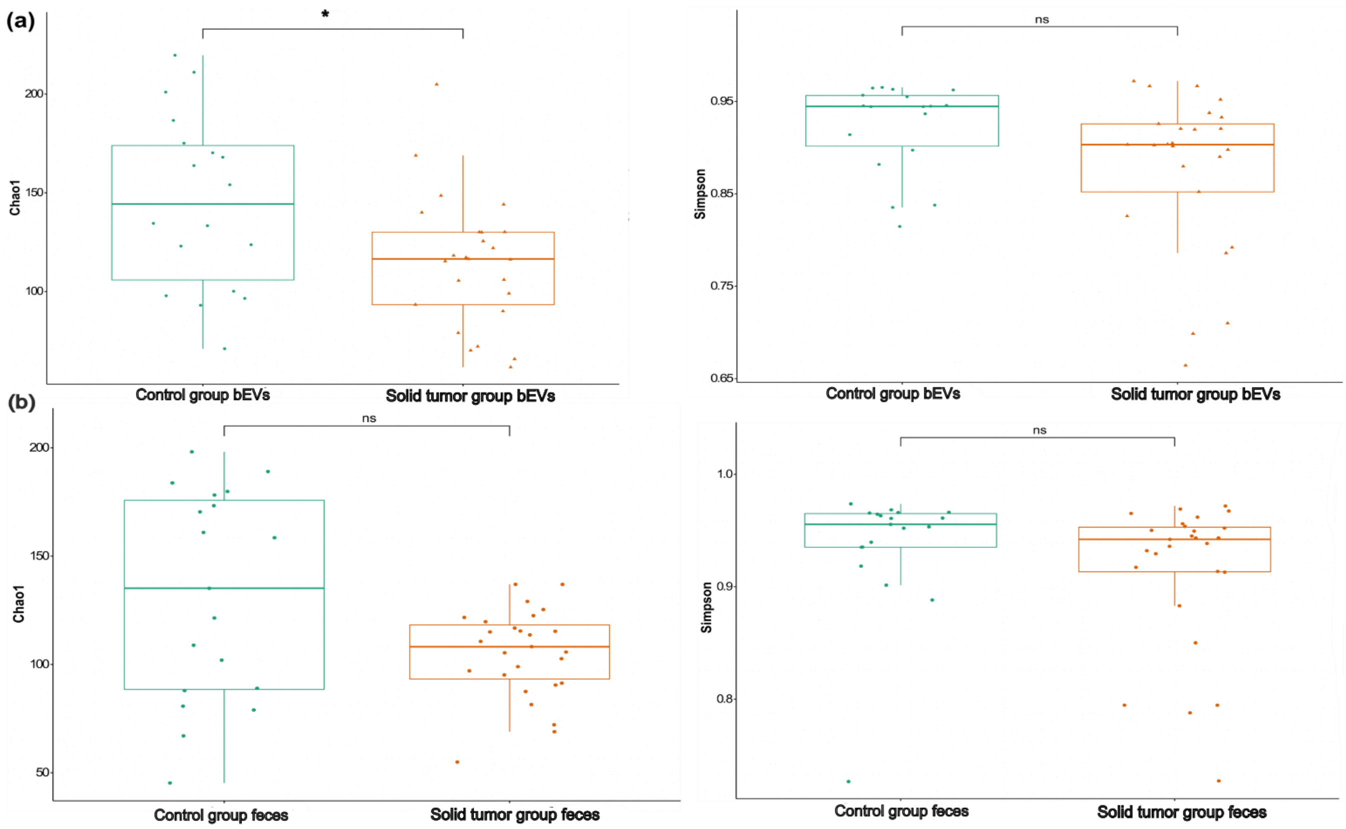

Supplementary Figure 4: Box plots depicting alpha diversity of gut microbiome-derived bacterial extracellular vesicles (bEVs) and whole feces, analyzed by Chao1 and Simpson index. Solid tumor patients show a decrease in bacterial richness and diversity, as observed in both bEVs and feces. (a) Alpha diversity and richness of gut microbiome-derived bacterial extracellular vesicles (bEVs) of healthy controls vs solid tumor patients (b) Alpha diversity and richness of gut of total fecal microbiota of healthy controls vs solid tumor patients. ns: $p > 0.05$; *: $p < 0.05$

Supplementary Table 1: A brief timeline of studies evaluating the role of microbiome-derived bEVs in human health and diseases.

Bacterial origin of EVs	Immune effect/health outcome	Reference
Bacteroides fragilis	EVs induce immunomodulatory effects upon administration and prevent experimental colitis.	Shen et al., 2012 [1]
Bifidobacterium bifidum LMG13195	Induce differentiation of T cells to regulatory T cells and IL-10 secretion; potential adjuvants for immunotherapy.	Lopez et al., 2012 [2]
Staphylococcus aureus	Inhalation of *S. aureus* EVs has been shown to cause airway inflammation *in vivo*.	Kim et al., 2012 [3]
Lactobacillus rhamnosus	Induce immunoregulatory effects and affect enteric nerve system *in vivo*.	Al-Nedawi et al., 2015 [4]
Streptococcus agalactiae	Disrupt the feto-maternal barrier and leading to preterm birth *in vivo*.	Surve et al., 2016 [5]
Bifidobacterium longum	Promote apoptosis of bone-marrow-derived mast cells and alleviate food allergy.	Kim et al., 2016 [6]
Helicobacter pylori	Induce inflammation and gastric cancer by secretion of inflammatory mediators from gastric epithelial cells.	Choi et al., 2017 [7]
kefir-derived Lactobacilli	Decrease the inflammatory response of human colorectal adenocarcinoma cell lines *in vitro*, protects from colitis.	Seo et al., 2018 [8]
Staphylococcus aureus	Increase secretion of proinflammatory cytokines, cause pneumonia.	An Y et al., 2019 [9]
Helicobacter pylori	Increase expression of proinflammatory cytokines, increase reactive oxygen species (ROS) and cause atherosclerosis	Wang et al., 2021 [10]
Fusobacterium nucleatum	Increase secretion of proinflammatory cytokines, cause intestinal inflammation.	Engevik et al., 2021 [11]
	Increase proinflammatory cytokines secretion, decreasing anti-inflammatory cytokines secretion, activation of autophagy and cause colitis	Wei S et al., 2023 [12]

Supplementary Table 2: Clinical characteristics of solid tumor patients

Sample ID	Location of solid tumor/neoplasm	Sample ID	Location of solid tumor/neoplasm
ST1	Overlapping lesion of lung	ST15	Upper lobe, lung<br>Lingula of lung<br>Upper lobe, bronchus
ST2	Lung, NOS	ST16	Malignant neoplasm of kidney, except renal pelvis
ST3	NA	ST17	Malignant neoplasm of kidney, except renal pelvis
ST4	Overlapping lesion of lung	ST18	Lower lobe, lung<br>Lower lobe, bronchus
ST5	Overlapping lesion of lung	ST19	Overlapping lesion of lung
ST6	Malignant melanoma of trunk	ST20	Malignant neoplasm of ureter
ST7	Malignant melanoma of trunk	ST21	Upper lobe, lung<br>Lingula of lung<br>Upper lobe, bronchus
ST8	Lower lobe, lung<br>Lower lobe, bronchus	ST22	Upper lobe, lung<br>Lingula of lung<br>Upper lobe, bronchus
ST9	Malignant neoplasm of kidney, except renal pelvis	ST23	Malignant melanoma of trunk
ST10	Malignant melanoma of trunk	ST24	Lower lobe, lung<br>Lower lobe, bronchus
ST11	Malignant neoplasm of kidney, except renal pelvis	ST25	Upper lobe, lung<br>Lingula of lung<br>Upper lobe, bronchus
ST12	Malignant neoplasm of kidney, except renal pelvis	ST26	Overlapping lesion of lung
ST13	Ventral surface of tongue, NOS	ST27	Lower lobe, lung<br>Lower lobe, bronchus
ST14	Malignant melanoma of trunk (Anal: margin, skin, Perianal skin, Skin of breast)	ST28	Lung, NOS Bronchus, NOS<br>Bronchiole<br>Bronchogenic<br>Pulmonary, NOS

Supplementary Table 3: Top 25 GO classes of bacterial proteins identified in bEVs from solid tumor patients and healthy controls using UniProtKB trEMBL and UniProtKB Swissprot databases

(a) GO classes of bacterial proteins based on biological process in solid tumor patients

GO protein class	Frequency
Amino acid metabolic process [GO:0006520]	4438
Carbohydrate metabolic process [GO:0005975]	3057
Protein metabolic process [GO:0019538]	1848
Carboxylic acid metabolic process [GO:0019752]	1175
Protein transport [GO:0015031]	1098
Ion transport [GO:0006811]	1087
Tricarboxylic acid cycle [GO:0006099]	572
Cellular iron ion homeostasis [GO:0006879]	571
Ion transmembrane transport [GO:0034220]	543
Lipid metabolic process [GO:0006629]	532
Cellular component organization or biogenesis [GO:0071840]	512
Phosphorylation [GO:0016310]	476
Response to stimulus [GO:0050896]	469
Organic substance metabolic process [GO:0071704]	88
One-carbon metabolic process [GO:0006730]	279
Nucleic acid metabolic process [GO:0090304]	270
Nucleotide metabolic process [GO:0009117]	203
Signal transduction [GO:0007165]	202
Cellular response to stimulus [GO:0051716]	186
Polysaccharide catabolic process [GO:0000272]	181
Peptide metabolic process [GO:0006518]	87
Carbohydrate transport [GO:0008643]	79
Carbohydrate derivative metabolic process [GO:1901135]	77
Metabolic process [GO:0008152]	70
Nitrogen compound metabolic process [GO:0006807]	38

(b) GO classes of bacterial proteins based on biological process in healthy controls

**GO protein class**	**Frequency**
**Amino acid metabolic process [GO:0006520]**	1661
Carbohydrate metabolic process [GO:0005975]	667
Ion transport [GO:0006811]	490
Protein transport [GO:0015031]	346
Cellular iron ion homeostasis [GO:0006879]	197
Protein metabolic process [GO:0019538]	196
Ion transmembrane transport [GO:0034220]	176
Phosphorylation [GO:0016310]	169
Protein folding [GO:0006457]	138
Organic substance metabolic process [GO:0071704]	124
Lipid metabolic process [GO:0006629]	118
Cellular component organization or biogenesis [GO:0071840]	102
Carboxylic acid metabolic process [GO:0019752]	90
Carbohydrate transport [GO:0008643]	59
Polysaccharide catabolic process [GO:0000272]	56
Peptide metabolic process [GO:0006518]	54
Nucleotide metabolic process [GO:0009117]	52
Cellular response to stimulus [GO:0051716]	41
Tricarboxylic acid cycle [GO:0006099]	34
One-carbon metabolic process [GO:0006730]	31
Nucleic acid metabolic process [GO:0090304]	14
Carbohydrate derivative metabolic process [GO:1901135]	9
Response to stimulus [GO:0050896]	9
Nitrogen compound metabolic process [GO:0006807]	8
Xenobiotic transport [GO:0042908]	6

(c) GO classes of bacterial proteins based on molecular function in solid tumor patients

**GO protein class**	**Frequency**
**Nucleotide binding [GO:0000166]**	7871
Oxidoreductase activity [GO:0016491]	6030
Structural molecule activity [GO:0005198]	3416
Metal ion binding [GO:0046872]	2875
Isomerase activity [GO:0016853]	2354
Transferase activity [GO:0016740]	1661
Transmembrane transporter activity [GO:0022857]	1568
ATP-dependent activity [GO:0140657]	1432
Peptidase activity [GO:0008233]	1233
RNA binding [GO:0003723]	1218
Protein binding [GO:0005515]	1109
Hydrolase activity [GO:0016787]	1027
Lyase activity [GO:0016829]	813
Ligase activity [GO:0016874]	648
Carbohydrate binding [GO:0030246]	593
Carbohydrate derivative binding [GO:0097367]	426
Carbohydrate transmembrane transporter activity [GO:0015144]	414
Lipid binding [GO:0008289]	353
DNA binding [GO:0003677]	298
Small molecule binding [GO:0036094]	273
Transmembrane signaling receptor activity [GO:0004888]	196
Molecular carrier activity [GO:0140104]	153
Nucleic acid binding [GO:0003676]	115
Iron-sulfur cluster binding [GO:0051536]	47
Ribosome binding [GO:0043022]	21

(d) GO classes of bacterial proteins based on molecular function in healthy controls

**GO protein class**	**Frequency**
**Nucleotide binding [GO:0000166]**	2118
Oxidoreductase activity [GO:0016491]	1532
Structural molecule activity [GO:0005198]	1371
Metal ion binding [GO:0046872]	874
Transmembrane transporter activity [GO:0022857]	820
Hydrolase activity [GO:0016787]	711
Isomerase activity [GO:0016853]	682
Transferase activity [GO:0016740]	413
Lyase activity [GO:0016829]	356
ATP-dependent activity [GO:0140657]	217
Protein binding [GO:0005515]	210
Carbohydrate binding [GO:0030246]	175
Lipid binding [GO:0008289]	149
Carbohydrate transmembrane transporter activity [GO:0015144]	106
Carbohydrate derivative binding [GO:0097367]	84
Serine-type endopeptidase inhibitor activity [GO:0004867]	42
DNA binding [GO:0003677]	39
Ligase activity [GO:0016874]	36
RNA binding [GO:0003723]	28
Nucleic acid binding [GO:0003676]	20
Iron-sulfur cluster binding [GO:0051536]	9
Virus receptor activity [GO:0001618]	6
Small molecule binding [GO:0036094]	6
Protein-macromolecule adaptor activity [GO:0030674]	1
Transferrin receptor activity [GO:0004998]	1

Supplementary Table 4: Ten most abundant bacterial taxa in gut microbiome-derived bacterial extracellular vesicles (bEVs) and total feces of solid tumor patients and healthy controls, obtained from 16S rRNA sequence using QIIME2

(a) Bacterial taxa in gut microbiome-derived bEVs of solid tumor patients and healthy controls

**Phylum**	bEVs solid tumor patients (%)	bEVs healthy controls (%)	Genus	bEVs solid tumor patients (%)	bEVs healthy controls (%)
Bacteroidota	51.1	41.9	*Bacteroides*	27.4	26.3
Firmicutes	34.1	43.9	*Alistipes*	14.9	7.4
Actinobacteriota	8.2	8.9	*Streptococcus*	1.1	9.5
Proteobacteria	5.9	4.9	*Prevotella_9*	4.0	4.6
Fusobacteriota	3.5	0.1	*Rhodococcus*	6.4	0.03
Acidobacteriota	0.1	0.08	*Izemoplasmatales*	3.4	2.9
Myxococcota	0.1	0	*Faecalibacterium*	2.8	2.8
Verrucomicrobiota	0.008	0.08	*Staphylococcus*	0.9	5.3
Spirochaetota	0.007	0.04	*[Eubacterium]_siraeum_group*	2.9	1.0
Deinococcota	0.017	0.01	*Cutibacterium*	4.1	4.1

(b) Bacterial taxa in the total feces of solid tumour patients and healthy controls

**Phylum**	**Feces solid tumor patients (%)**	**Feces healthy controls (%)**	**Genus**	**Feces solid tumor patients (%)**	**Feces healthy controls (%)**
Bacteroidota	59.2	52.7	*Bacteroides*	36.9	30.4
Firmicutes	37.2	40.3	*Alistipes*	9.6	8.7
Proteobacteria	2.9	4.9	*Faecalibacterium*	4.1	7.3
Actinobacteriota	0.1	1.9	*Sutterella*	2.3	3.6
Fusobacteriota	0.6	0.01	*Parabacteroides*	2.6	2.6
Verrucomicrobiota	0.02	0.07	*RF39*	2.2	2.9
Deinococcota	0	0.04	*Paraprevotella*	3.2	0.9
Acidobacteriota	0	0.03	*Ruminococcus*	1.9	1.5
Planctomycetota	0	0.02	*Blautia*	1.9	1.5
Chloroflexi	0	0.01	*Agathobacter*	1.7	1.5

References (Supplementary Table 1):

[1] Shen Y, Torchia MLG, Lawson GW, Karp CL, Ashwell JD, Mazmanian SK. Outer Membrane Vesicles of a Human Commensal Mediate Immune Regulation and Disease Protection. Cell Host Microbe 2012;12:509–20. https://doi.org/10.1016/j.chom.2012.08.004.

[2] López P, González-Rodríguez I, Sánchez B, Gueimonde M, Margolles A, Suárez A. Treg-inducing membrane vesicles from Bifidobacterium bifidum LMG13195 as potential adjuvants in immunotherapy. Vaccine 2012;30:825–9. https://doi.org/10.1016/j.vaccine.2011.11.115.

[3] Kim M -R., Hong S -W., Choi E -B., Lee W -H., Kim Y -S., Jeon SG, et al. *<scp>S</scp> taphylococcus aureus* -derived extracellular vesicles induce neutrophilic pulmonary inflammation via both <scp>T</scp> h1 and <scp>T</scp> h17 cell responses. Allergy 2012;67:1271–81. https://doi.org/10.1111/all.12001.

[4] Al-Nedawi K, Mian MF, Hossain N, Karimi K, Mao Y, Forsythe P, et al. Gut commensal microvesicles reproduce parent bacterial signals to host immune and enteric nervous systems. The FASEB Journal 2015;29:684–95. https://doi.org/10.1096/fj.14-259721.

[5] Surve MV, Anil A, Kamath KG, Bhutda S, Sthanam LK, Pradhan A, et al. Membrane Vesicles of Group B Streptococcus Disrupt Feto-Maternal Barrier Leading to Preterm Birth. PLoS Pathog 2016;12:e1005816. https://doi.org/10.1371/journal.ppat.1005816.

[6] Kim J-H, Jeun E-J, Hong C-P, Kim S-H, Jang MS, Lee E-J, et al. Extracellular vesicle–derived protein from Bifidobacterium longum alleviates food allergy through mast cell suppression. Journal of Allergy and Clinical Immunology 2016;137:507-516.e8. https://doi.org/10.1016/j.jaci.2015.08.016.

[7] Choi H-I, Choi J-P, Seo J, Kim BJ, Rho M, Han JK, et al. Helicobacter pylori-derived extracellular vesicles increased in the gastric juices of gastric adenocarcinoma patients and induced inflammation mainly via specific targeting of gastric epithelial cells. Exp Mol Med 2017;49:e330–e330. https://doi.org/10.1038/emm.2017.47.

[8] Seo MK, Park EJ, Ko SY, Choi EW, Kim S. Therapeutic effects of kefir grain Lactobacillus-derived extracellular vesicles in mice with 2,4,6-trinitrobenzene sulfonic acid-induced inflammatory bowel disease. J Dairy Sci 2018;101:8662–71. https://doi.org/10.3168/jds.2018-15014.

[9] An Y, Wang Y, Zhan J, Tang X, Shen K, Shen F, et al. Fosfomycin Protects Mice From Staphylococcus aureus Pneumonia Caused by α-Hemolysin in Extracellular Vesicles by Inhibiting MAPK-Regulated NLRP3 Inflammasomes. Front Cell Infect Microbiol 2019;9. https://doi.org/10.3389/fcimb.2019.00253.

[10] Wang N, Zhou F, Chen C, Luo H, Guo J, Wang W, et al. Role of Outer Membrane Vesicles From Helicobacter pylori in Atherosclerosis. Front Cell Dev Biol 2021;9. https://doi.org/10.3389/fcell.2021.673993.

[11] Engevik MA, Danhof HA, Ruan W, Engevik AC, Chang-Graham AL, Engevik KA, et al. *Fusobacterium nucleatum* Secretes Outer Membrane Vesicles and Promotes Intestinal Inflammation. MBio 2021;12. https://doi.org/10.1128/mBio.02706-20.

[12] Wei S, Zhang J, Wu X, Chen M, Huang H, Zeng S, et al. *Fusobacterium nucleatum* Extracellular Vesicles Promote Experimental Colitis by Modulating Autophagy via the miR-574-5p/CARD3 Axis. Inflamm Bowel Dis 2023;29:9–26. https://doi.org/10.1093/ibd/izac177.
